# Bornyl Diphosphate Synthase From *Cinnamomum burmanni* and Its Application for (+)-Borneol Biosynthesis in Yeast

**DOI:** 10.3389/fbioe.2021.631863

**Published:** 2021-02-11

**Authors:** Rui Ma, Ping Su, Juan Guo, Baolong Jin, Qing Ma, Haiyan Zhang, Lingli Chen, Liuying Mao, Mei Tian, Changjiangsheng Lai, Jinfu Tang, Guanghong Cui, Luqi Huang

**Affiliations:** ^1^State Key Laboratory Breeding Base of Dao-di Herbs, National Resource Center for Chinese Materia Medica, China Academy of Chinese Medical Sciences, Beijing, China; ^2^School of Pharmacy, Henan University of Chinese Medicine, Zhengzhou, China; ^3^Department of Chemistry, The Scripps Research Institute, Jupiter, FL, United States

**Keywords:** (+)-borneol, (+)-bornyl diphosphate synthase, *Cinnamomum burmanni*, metabolic engineering, *Saccharomyces cerevisiae*

## Abstract

(+)-Borneol is a desirable monoterpenoid with effective anti-inflammatory and analgesic effects that is known as soft gold. (+)-bornyl diphosphate synthase is the key enzyme in the (+)-borneol biosynthesis pathway. Despite several reported (+)-bornyl diphosphate synthase genes, relatively low (+)-borneol production hinders the attempts to synthesize it using microbial fermentation. Here, we identified the highly specific (+)-bornyl diphosphate synthase CbTPS1 from *Cinnamomum burmanni*. An *in vitro* assay showed that (+)-borneol was the main product of CbTPS1 (88.70% of the total products), and the *K*_*m*_ value was 5.11 ± 1.70 μM with a *k*_*cat*_ value of 0.01 s^–1^. Further, we reconstituted the (+)-borneol biosynthetic pathway in *Saccharomyces cerevisiae*. After tailored truncation and adding Kozak sequences, the (+)-borneol yield was improved by 96.33-fold to 2.89 mg⋅L^–1^ compared with the initial strain in shake flasks. This work is the first reported attempt to produce (+)-borneol by microbial fermentation. It lays a foundation for further pathway reconstruction and metabolic engineering production of this valuable natural monoterpenoid.

## Introduction

The monoterpene borneol is a highly desirable natural product widely used in medicine, spice, and chemical fields since ancient times (Wojtunik-Kulesza et al., 2019). It has a broad spectrum of bidirectional regulation on the central nervous system (Zhang et al., 2017; Zheng et al., 2018); anti-inflammatory (Zou et al., 2017; Ji et al., 2020) and antimicrobial activities (Xin et al., 2020); and increases biofilm barrier permeability (Song et al., 2018; Chen et al., 2019). Borneol is divided into (+)-borneol and (−)-borneol according to optical rotations. Natural (+)-borneol has primarily been extracted from *Cinnamomum camphora* (L.) Presl and *C. burmanni* (Nees et T.Nees) Blume (borneol-type) since the 1980s in China (Chen et al., 2010). However, the slow growth rate, low (+)-borneol levels, and restricted cultivation area mean that the yield of natural (+)-borneol is far from meeting the market demand. Borneol synthesized by chemical methods thus occupies most of the market share; however, a certain number of toxic compounds, such as isoborneol may exist in synthetic borneol. Thus, it is necessary to explore other methods to produce natural (+)-borneol.

**FIGURE 1:**
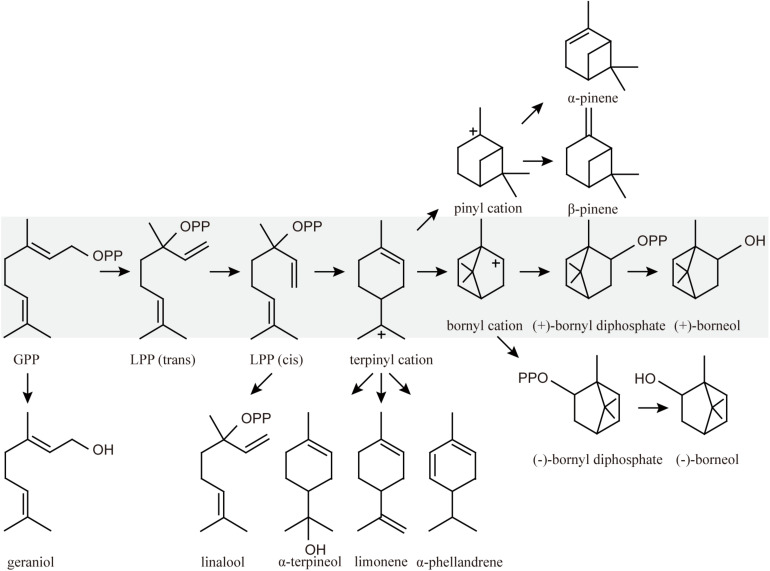
Proposed mechanism for BPPS. The primary pathway leads to the formation of borneol (gray) and other monoterpenoid products.

Due to the clear genetic background and lack of susceptibility to phage infections, *Saccharomyces cerevisiae* is the preferred host for metabolic engineering (Kirby and Keasling, 2009; Liu et al., 2019; Nielsen, 2019). Many monoterpenoids, such as geraniol, limonene, linalool, and α-terpineol (Figure 1) have been produced in *S. cerevisiae* (Amiri et al., 2016; Cao et al., 2016, 2017; Zhang et al., 2019, 2020). Isopentenyl diphosphate (IPP) and its isomer dimethylallyl diphosphate (DMAPP) derived from the mevalonate pathway (MVA) are the precursors of all terpenoids in *S. cerevisiae*, and geranyl diphosphate (GPP) is the direct precursor of monoterpenes catalyzed by farnesyl diphosphate synthase (ERG20) (Jiang et al., 2017). Thus, in order to produce monoterpenes in yeast, ERG20 is usually mutated or rationally designed into GPP synthase (Ignea et al., 2014; Zhao et al., 2016; Jiang et al., 2017). The upstream MVA pathway genes, *tHMG1* and *IDI1*, are frequently overexpressed in yeast (Zhao et al., 2016; Zhang et al., 2019). Guo et al. (2018) even overexpressed all MVA pathway genes (*ERG10*, *ERG13*, *tHMG1*, *ERG12*, *ERG8*, *ERG19*, *IDI1*, *ERG20*) to increase the GPP pool. Modification of the monoterpene synthase, including translational fusion and truncation of transit peptides at the N-terminus of the enzymes, is also an effective strategy for increasing the production of terpenes (Jongedijk et al., 2015; Ignea et al., 2019; Hu et al., 2020).

Like other monoterpenes, bornyl diphosphate synthase (BPPS) is the key enzyme involved in (+)-borneol biosynthesis. It catalyzes the universal precursor GPP to form (+)-bornyl diphosphate, and is then dephosphorylated to produce the target product (+)-borneol (Figure 1). BPPSs have been identified from several plants, including *Salvia officinalis* (SBS), *Lavandula angustifolia* (LaBPPS), *Lippia dulcis* (LdBPPS), and *Amomum villosum* (AvBPPS) (Wise et al., 1998; Despinasse et al., 2017; Hurd et al., 2017; Wang et al., 2018). However, all these enzymes produced multiple products, such as α-pinene, β-pinene, camphene, and limonene, with the largest amount of (+)-borneol produced by SBS, accounting for 57.8% of the total products. However, there have been no attempts to produce this valuable product by microbial cell factories.

Here we report a high-specificity (+)-borneol BBPS gene (*CbTPS1*) from *C. burmanni*. Among the products with GPP as substrate in an *in vitro* assay, (+)-borneol accounted for 88.70% of the total. We thus aimed to construct a (+)-borneol biosynthesis pathway in *S. cerevisiae*. To reach the target, eight genes involved in the MVA pathway were overexpressed. Truncated transit peptides and adding the Kozak sequence of CbTPS1 further improved the (+)-borneol production. Our work provides a good example for (+)-borneol production in microbial fermentation.

## Materials and Methods

### Plant Materials and Chemicals

Leaves of *Cinnamomum burmanni* (Nees et T.Nees) Blume were obtained from Guangdong Huaqingyuan Technology Co., Ltd. Plant leaf material grown in natural conditions was picked in May 2019. *C. burmannii* was identified by Prof. Cui Guanghong of China Academy of Chinese Medical Sciences and stored at −80°C for further usage (Storage Number: YXS201905). GPP, geraniol, α-pinene, β-pinene, α-phellandrene, limonene, α-terpineol, (+)-borneol and (−)-borneol standards were purchased from Sigma-Aldrich Chemical Co., United States.

### RNA Extraction, cDNA Synthesis

The total RNA from *C. burmannii* leaves was extracted using a quick RNA isolation kit (HuaYueYang Biotechnology, China) based on the manufacturer’s protocol, and then digested and purified by RNase-free DNase I (TaKaRa, Japan). An aliquot containing 1 μg total RNA was used to synthesize the first-strand cDNA with TransScript One-Step gDNA Removal and cDNA Synthesis SuperMix (TransGen Biotechnology, China) according to the manufacturer’s guidelines.

### BPPS Candidate Selection and Analysis

Transcriptomic libraries of the *C. burmannii* leaves were shipped to the Novogene Company^1^ for library construction and RNA-seq. The Illumina-derived nucleotide sequences reported in this paper have been submitted to China National Center for Bioinformation^2^ under accession number CRA003558. To mine the BPPS candidate genes, TBLASTN analysis of BPPSs in the *C. burmannii* transcriptome was carried out using BioEdit software (Su et al., 2018). *SBS* (GenBank Accession Number: AAC26017), *LaBPPS* (GenBank Accession Number: AJW68082), *LdBPPS* (GenBank Accession Number: ATY48638), and *AvBPPS* (GenBank Accession Number: AWW87313) were used as the query sequences. The *CbTPS1* (GenBank Accession Number: MW196671) sequence was analyzed using NCBI^3^. The open reading frames (ORFs) were identified using the ORF Finder^4^, and deduced amino acid sequences were identified using ExPASy^5^. Multiple sequence alignments were conducted using CLC Bio Sequence Viewer 6^6^. The chloroplast transit peptide of CbTPS1 was predicted by ChloroP^7^.

All statistical analyses were conducted using SPSS version 23.0 (SPSS Inc., Chicago, IL, United States) for windows. One-way analysis of variance was used to compare the mean difference in (+)-borneol of strains. The *P*-value of less than 0.05 considered statistically significant.

### Gene Cloning, Protein Expression and Purification

The ORF was cloned using specially designed primers (Supplementary Table 1). Phusion High-Fidelity PCR Master Mix (New England BioLabs, United States) was used for amplification reaction according to the included protocol. PCR products were purified, and then ligated into the pEASY^®^-Blunt Simple Cloning Vector (TransGen Biotech, China) and transformed into *E. coli* DH5α cells. Positive colonies were verified by sequencing (Beijing RuiBo Biotechnology Co., Ltd., China) and then subcloned into the pET-32a (+) expression vector (Novagen, United States) according to the protocol of the pEASY^®^-Uni Seamless Cloning and Assembly Kit (TransGen Biotech, China) (Supplementary Table 1).

Recombinant proteins were expressed and purified following the methods described previously (Ma et al., 2020), with some modifications as follows: the 200 mL bacterial solution was centrifuged (5,000 × g, 5 min, 4°C) to collect the cell pellets, and resuspended in 5 ml assay buffer (50 mM HEPES, pH 7.2, 10 mM MgCl_2_, 5 mM dithiothreitol), and then a sonicator was used to lyse cells. The lysates were centrifuged (12,000 × g, 30 min, 4°C) to produce crude protein. And then the His-tagged purified proteins were eluted using a buffer equivalent to the binding buffer but supplemented with different concentrations of imidazole (50, 100, 250, 350, and 500 mM). Fractions containing the target protein were pooled together and concentrated to a volume of 1 mL using an Amicon Ultra-15 centrifugal filter unit with an Ultracel-30 membrane (Merck Millipore, Germany). Protein concentrations were determined using the Bradford Assay (Cowin Biotech, China). The protein samples were assessed by sodium dodecyl sulfate polyacrylamide gel electrophoresis (SDS-PAGE).

### *In vitro* Enzyme Assays and Kinetic Assays

*In vitro* enzyme assays followed the method described below: enzyme assays were performed in 300 μL, containing 50 mM HEPES (Ph 7.2), 10 mM MgCl_2_, 5 mM DTT, 1 mM PMSF, 380 nM of the enzyme and 50 μM GPP, incubated for 1 h at 30°C. Then 1.5 μL calf intestinal alkaline phosphatase (CIAP) (TaKaRa, Japan) was added, followed by incubation for 2 h at 37°C to allow enzymatic dephosphorylation. Time-course experiments were carried out to obtain the initial speed of the enzymatic reaction from 1 to 180 min (Supplementary Figure 1). Then, 3 min was used in the kinetic assays. The enzyme assays were performed in a 300 μL reaction volume at 30°C. A concentration that ranged from 0.125 to 150 μM GPP substrate was used. After 3 min incubation, the reaction was terminated at 80°C for 3 min, followed by quenching in ice, and then added 1.5 μL CIAP, followed by incubation for 30 min at 37°C. Assay products were extracted twice with 300 μL of hexane and samples were concentrated under a gentle nitrogen flow. The samples were then redissolved with 100 μL of hexane before analysis with gas chromatography coupled with mass spectrometry (GC-MS) (described below).

The GraphPad Prism version 5 for Windows (GraphPad Software, La Jolla California United States)^8^ was used to obtain kinetic parameters by fitting the obtained data to the Michaelis-Menten equation. All assays were performed in triplicate.

### Construction of (+)-Borneol Producing Strains

The initial strain used in this study was CEN.PK2-1D derived from *S. cerevisiae* (Table 1). All the endogenous genes (*ERG10*, *ERG13*, *tHMG1*, *ERG12*, *ERG8*, *ERG19*, *IDI1*, *ERG20*) involved in the MVA pathway were amplified from CEN.PK2-1D genomic DNA. The mutant of *ERG20*, *ERG20^*F*96*W–N*127*W*^*, used in this work was reported to possess higher efficiency for monoterpene production (Jiang et al., 2017). The M2S integration method was applied to integrate gene expression cassettes into the yeast chromosome (Li et al., 2016). Briefly, *ERG10* and *ERG13* were amplified with the addition of a *Bsa*I digestion site and ligated with head-to-head promoters (*pGAL1-pGAL10*) into the terminator vector T1-(TPI1-PGI1), resulting in the plasmid T1-(*ERG10-ERG13*). Two terminators were inserted into the scaffold plasmid, with dedicated homologous arms L1 and L2 lying on both sides. Similarly, plasmids T2-(*tHMG1-tHMG1*), T3-(*tHMG1-ERG12*), T4-(*ERG8-ERG19*), and T5-(*IDI1-ERG20^*F*96*W–N*127*W*^*) were generated with dedicated homologous arms L2 and L3, L3 and L4, L4 and L5, L5 and L6, respectively. Each expression cassette with designed homologous arms was amplified individually. The integration site *YPRC*Δ*15* was chosen as the target locus, and *URA3* was chosen as the selection marker. The upstream homologous arm *YPRC*Δ*15*-UP was amplified from CEN.PK2-1D genomic DNA; *URA3* cassette including the promoter was amplified from pESC-URA vector; and L1 arm was amplified from terminator vector T1. These three parts were assembled to form the selection marker module *YPRC*Δ*15*UP-URA3-L1 through overlap extension PCR. The downstream homologous arm *YPRC*Δ*15*DOWN was amplified from CEN.PK2-1D genomic DNA and the L6 arm was amplified from terminator vector T5, and they were then combined to generate the downstream homologous arm module L6-*YPRC*Δ*15*DOWN. All the amplified fragments were used to co-transform CEN.PK2-1D for assembly and integration, and transformants were selected on synthetic drop in medium-Ura (SD-Ura) containing 20 g⋅L^–1^ glucose and 18 g⋅L^–1^ agar. Positive transformants were verified by sequencing, yielding the strain MD.

**TABLE 1:** Information of strains and vectors used in this study.

Strains or vectors	Description	Source
CEN.PK2-1D	*MAT*α, *URA3-52, TRP1-289, LEU2-3112, HIS3*Δ*1, MAL2-8C, SUC2*	EUROSCARF
MD	CEN.PK2-1D, *YPRC△15 URA3-P_*GAL*__1_-ERG10-T_*TPI*__1_-P_*GAL*__10_-ERG13-T_*PGI*_-P_*GAL*__1_*-*tHMG1*-*T_*ADH*__1_*-*P_*GAL*__10_*-*tHMG1*-*T_*CYC*__1_-P_*GAL*__1_*-*tHMG1*-*T_*FBA*__1_*-*P_*GAL*__10_*-*ERG12*-*T_*PDC*__1_-P_*GAL*__1_*-*ERG8*-*T_*RPS*__2_*-*P_*GAL*__10_*-*ERG19*-*T_*TDH*__1_-P_*GAL*__1_*-*IDI1*-*T_*CCW*__12_*-*P_*GAL*__10_*-*ERG20^*F*96*W–N*127*W*^*-*T_*RPL*__9__*A*_*	This study
MD-1	MD, pESC-LEU::*CbTPS1*	This study
MD-2	MD, pESC-LEU::*CbTPS1K*	This study
MD-3	MD, pESC-LEU::*t10-CbTPS1*	This study
MD-4	MD, pESC-LEU::*t10-CbTPS1K*	This study
MD-5	MD, pESC-LEU::*t32-CbTPS1*	This study
MD-6	MD, pESC-LEU::*t32-CbTPS1K*	This study
MD-7	MD, pESC-LEU::*t37-CbTPS1*	This study
MD-8	MD, pESC-LEU::*t37-CbTPS1K*	This study
T1-(TPI1-PGI)	Terminator vector with terminators TPI1 and PGI	This study
T2-(ADH1-CYC1)	Terminator vector with terminators ADH1 and CYC1	This study
T3-(FBA1-PDC1)	Terminator vector with terminators FBA1 and PDC1	This study
T4-(RPS2-TDH1)	Terminator vector with terminators RPS2 and TDH1	This study
T5-(CCW12-RPL9A)	Terminator vector with terminators CCW12 and RPL9A	This study

For (+)-borneol production, the yeast codon-optimized CbTPS1 as well as three truncated variants of CbTPS1 (at positions S10, S32 and C37) were cloned into the *Bam*HI site of the pESC-Leu vector (Agilent Technologies, United States) according to the pEASY-Uni Seamless Cloning and Assembly Kit (TransGen Biotech, Beijing, China), yielding the plasmids pESC-LEU:*CbTPS1*, pESC-LEU:*t10-CbTPS1*, pESC-LEU:*t32-CbTPS1*, and pESC-LEU:*t37-CbTPS1*. Further, yeast-specific Kozak sequence was added in front of the START codon ATG of CbTPS1 and the three truncated variants, generating pESC-LEU:*CbTPS1K*, pESC-LEU:*t10-CbTPS1K*, pESC-LEU:*t32-CbTPS1K*, and pESC-LEU:*t37-CbTPS1K*. Plasmids with the correct sequence were transferred to the host strain MD using Frozen-EZ Yeast Transformation II^TM^ (Zymo Research, United States) to obtain the (+)-borneol producing strains (Table 1). All the primers used are listed in Supplementary Table 1.

### Shake Flask Fermentation

For shake flask fermentation, the positive strains were cultured in flasks (50 ml) containing 10 ml of synthetic drop-out medium without leucine and uracil (SD-Leu-Ura) (FunGenome, China) at 30°C and 200 rpm for 48 h. Next, the cells were collected and induced by GAL promoters in 10 ml of YPL (1% yeast extract, 2% peptone, and 2% galactose) medium at 30°C and 200 rpm for 48 h. The fermentation products were extracted with an equal volume of ethyl acetate for 1 h, and centrifuged at 13,000 × g for 10 min to separate the upper organic phase for analyzing by GC-MS (described below). The calibration curves for content determination are shown in Supplementary Figure 2. All assays were performed in triplicate.

### Analysis Using GC-MS

The assay was carried out using a Trace 1310 series GC with a TSQ8000 MS detector (Thermo Fisher Scientific, United States). A TR-5 ms capillary column (30 m × 0.25 mm i.d., 0.25 μm film thickness; Thermo Fisher Scientific, United States) was used. The carrier gas for GC was helium at a flow rate of 1.0 mL⋅min^–1^. The oven program was as follows: 50°C for 2 min, linear ramp up at a rate of 5°C⋅min^–1^ to 230°C, held at 230°C for 5 min, followed with a linear ramp up at a rate of 10°C⋅min^–1^ to 300°C, held at 300°C for 2 min. The injector temperature and transfer line temperature were 280°C.

A chiral column, Agilent CycloSil-B (30 m × 0.25 mm i.d., 0.25 μm film thickness), was used to identify the chirality of the assay product and the content of borneol and camphor in *C. camphora* leaves. The carrier gas for GC was helium at a flow rate of 1.0 mL⋅min^–1^. The oven program was as follows: 50°C for 2 min, followed by a gradient from 50°C to 180°C at 5°C⋅min^–1^, then 10°C⋅min^–1^ to 230°C, held at 230°C for 2 min. The injector temperature was 200°C, and the transfer line temperature was 230°C.

## Results

### Transcriptome-Based Discovery of (+)-Bornyl Diphosphate Synthase in 2

Based on the high abundance of (+)-borneol in the leaves of *C. burmanni* (Shi et al., 2013), we used RNA isolated from young leaves to produce the transcriptome sequences. The reported BPPS genes were further queried against the *de novo* assembly of these sequences, showing that trinity_1267_c0_g1_i1 had the highest identity with all the reported genes. Trinity_1267_c0_g1_i1 was present as full-length sequence. It was further cloned using specific primers and annotated as CbTPS1.

**FIGURE 2:**
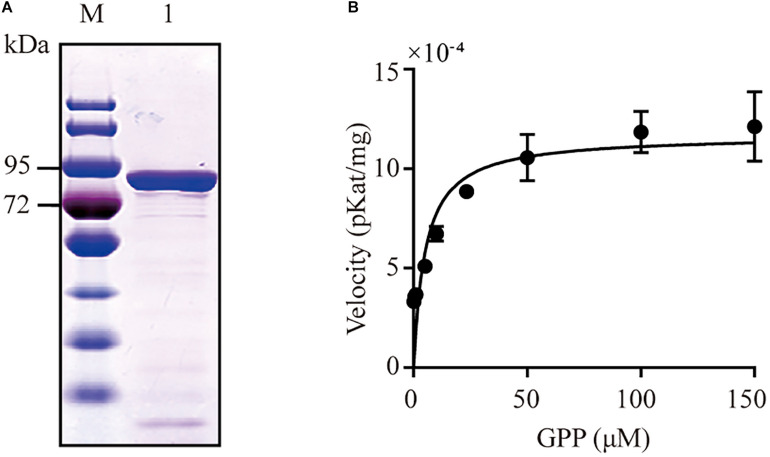
SDS-PAGE and kinetic assays analysis of CbTPS1. **(A)** SDS-PAGE of CbTPS1 *in vitro* assays. **(B)** Velocity of CbTPS1 at increasing GPP concentrations.

**FIGURE 3:**
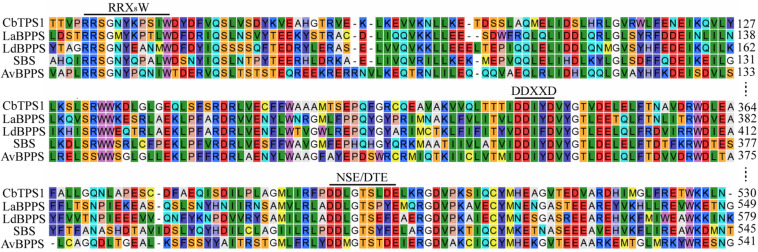
Partial alignment of CbTPS1 protein sequence with those of other known BPPSs. LaBPPS (accession No. AJW68082); LdBPPS (accession No. ATY48638); SBS (accession No. AAC26017); AvBPPS (accession No. AWW87313). The conserved RRX_8_W, DDXXD and NSE/DTE motifs are underlined.

CbTPS1 has an open reading frame of 1,812 bp that encodes a 603-residue enzyme with a calculated molecular mass of 69.1 kDa (Figure 2A). It was classified into the TPS-b subfamily, which contains three motifs of typical terpene synthases, namely the RRX_8_W motif responsible for monoterpenoid cyclization (Chen et al., 2011); and the DDXXD and NSE/DTE motifs in the C-terminal domain, which are responsible for metal-dependent ionization and substrate binding (Chen et al., 2011). Homologous alignment analysis showed that CbTPS1 shared highest sequence identities with SBS (41.75%) from *Salvia officinalis* (Wise et al., 1998; Figure 3), followed by AvBPPS (40.10%) from *Amomum villosum* (Wang et al., 2018), LaBPPS (38.68%) from *Lavandula angustifolia* (Despinasse et al., 2017), and LdBPPS (36.20%) from *Lippia dulcis* (Hurd et al., 2017).

### Functional Analysis of CbTPS1

**FIGURE 4:**
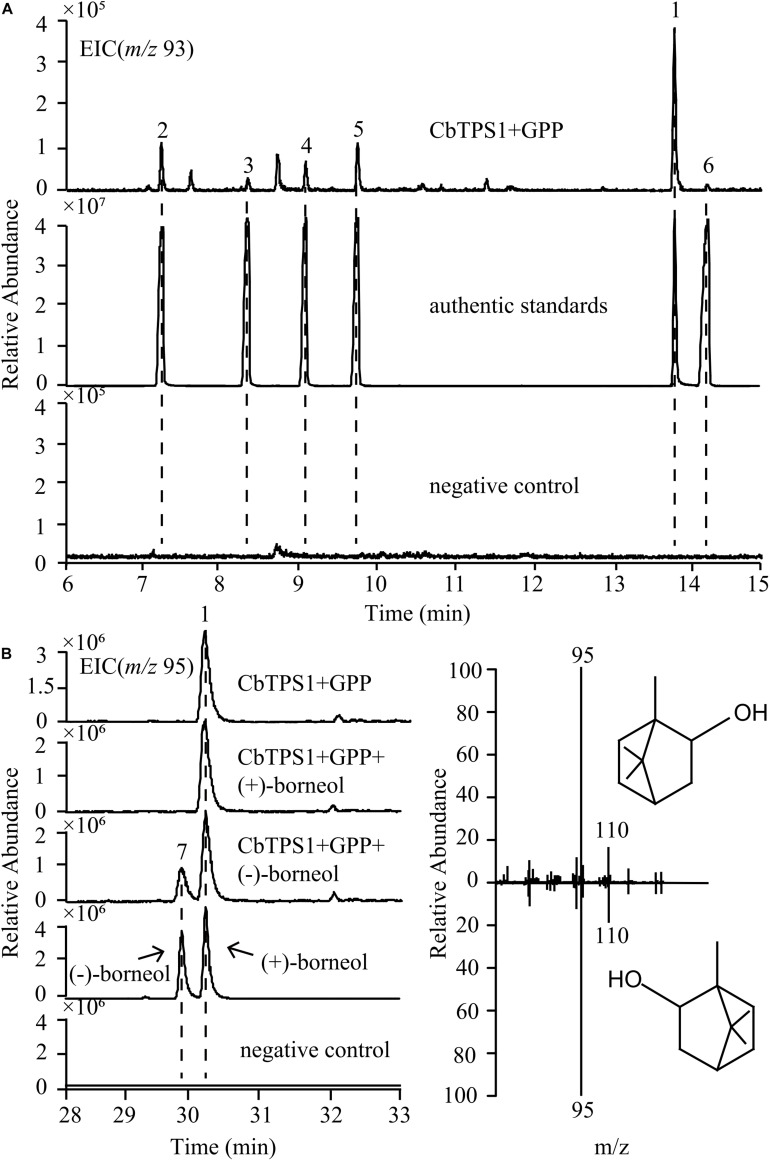
GC-MS analysis of *in vitro* assays with CbTPS1. **(A)** Extracted ion chromatograms of *m/z* 93 *in vitro* assays with purified CbTPS1 and GPP as a substrate. Peak 1, (+)-borneol, Peak 2, α-pinene, Peak 3, β-pinene, Peak 4, α-phellandrene, Peak 5, limonene, Peak 6, α-terpineol. **(B)** Chromatogram of borneol product compared with authentic standards (+)- and (−)-borneol. Peak 1, (+)-borneol, Peak 7, (−)-borneol. Corresponding mass spectrum of (+)-borneol (upper halves) and (−)-borneol (lower halves). EIC, Extracted ion chromatograms.

The recombinant protein of CbTPS1 was expressed in *E. coli* Transetta (DE3) cells using the pET-32a (+) expression vector, and then its function was identified with GPP as a substrate. CIAP was then added to remove the diphosphate group from the intermediate product. The purified CbTPS1 produced several monoterpenes (Figure 4A). Borneol was predominant (88.70%) with small amounts of α-pinene (2.70%), β-pinene (0.76%), α-phellandrene (1.20%), limonene (2.37%), and other minor monoterpenoids (4.27%). CbTPS1 was further examined for its catalytic properties and the *K*_*m*_ value was 5.11 ± 1.70 μM with a *k*_*cat*_ value of 0.01 s^–1^ (Figure 2B). In parallel, no product formation was found when the empty vector was transformed into *E. coli* Transetta (DE3) cells, and no product was produced in the absence of CIAP.

A chiral column was used to identify the chirality of borneol. Based on the results of GC-MS analysis (Figure 4B), a single product (peak 1) corresponding to the authentic standard (+)-borneol was detected. When the authentic standard (+)-borneol was added to the reaction product, only peak 1 was detected. However, a new product (peak 7) was detected when the authentic standard (−)-borneol was added. This result further proved that (+)-borneol was produced with GPP as a substrate.

### Reconstituting the MVA Pathway in Yeast for (+)-Borneol Production

**FIGURE 5:**
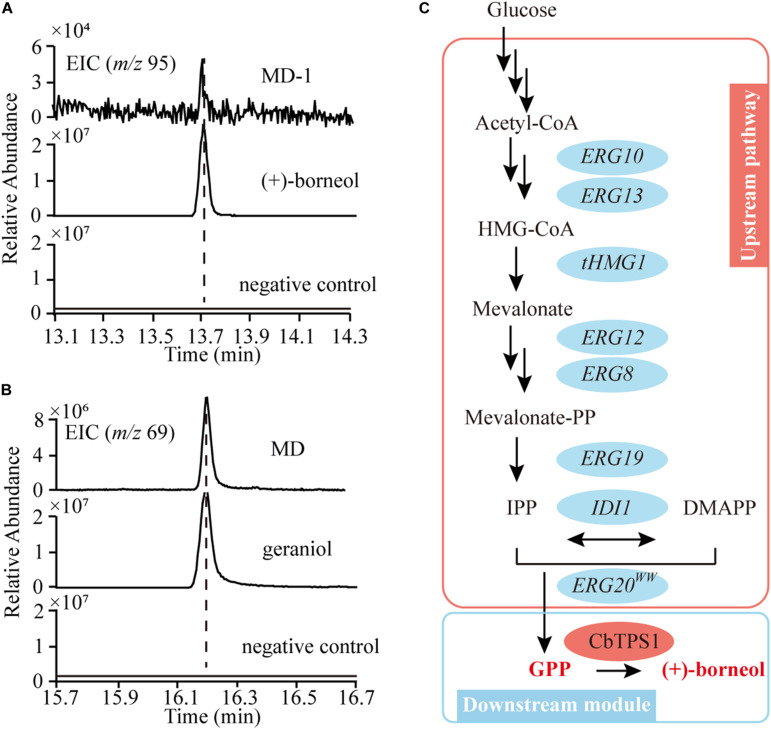
The biosynthetic pathway of GPP and (+)-borneol in *S. cerevisiae*. **(A)** Extracted ion chromatograms of *m/z* 95 of (+)-borneol production in CEN.PK2-1D (negative control) and the MD-1 strain. **(B)** Extracted ion chromatograms of *m/z* 69 of geraniol production in CEN.PK2-1D (negative control) and the MD strain. **(C)** Reconstitution of the MVA pathway in yeast for GPP production (pink ellipses), and the biosynthetic pathway of (+)-borneol (blue ellipses).

When the codon-optimized CbTPS1 was overexpressed in yeast CEN.PK2-1D, (+)-borneol could not be detected (Figure 5A). In addition, geraniol (the dephosphorylated GPP, precursor of (+)-borneol) was not detected in CEN.PK2-1D (Figure 5B). Hence, we reconstituted the MVA pathway in CEN.PK2-1D by overexpressing all the MVA pathway genes (*ERG10*, *ERG13*, *tHMG1*, *ERG12*, *ERG8*, *ERG19*, *IDI1*, *ERG20^*F*96*W–N*127*W*^*) to increase the precursor pool (Figure 5C). The obtained chassis strain MD can accumulate 12.52 mg⋅L^–1^ geraniol (Figure 5B). Then CbTPS1 was overexpressed in strain MD, and (+)-borneol was generated with a yield of 0.03 mg⋅L^–1^ (strain MD-1) (Figure 5A).

### Improving the (+)-Borneol Yield by Tailored Truncations

**FIGURE 6:**
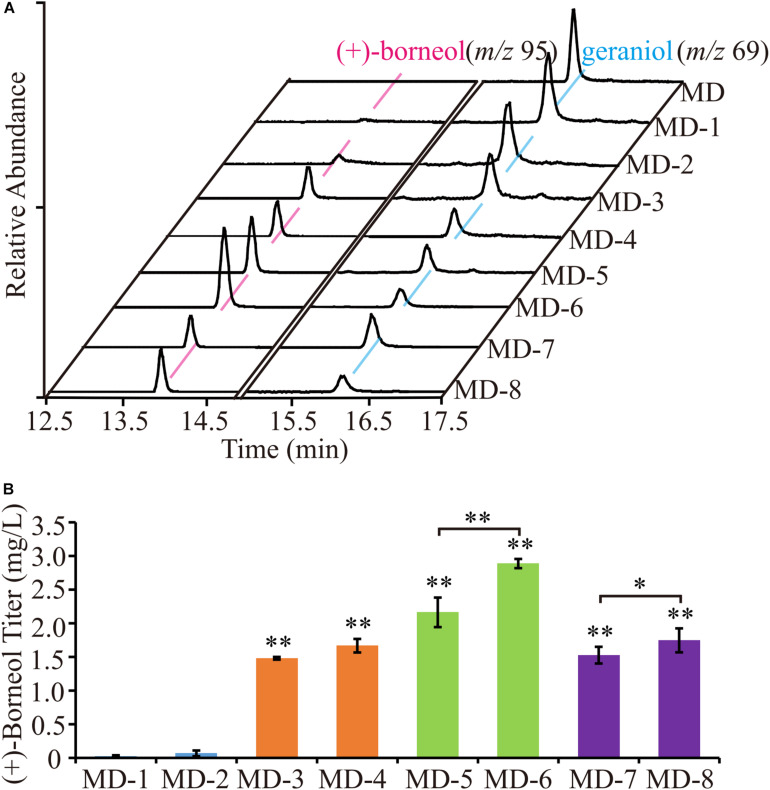
The (+)-borneol production of strains expressing truncations of the CbTPS1. **(A)** GC-MS analysis of the fermentation products of strains expressing the truncated proteins. (**B**) The titer of (+)-borneol product in strains expressing the truncated proteins. (^∗^) represent means which are significantly different at *p* < 0.05; (^∗∗^) represent means which are significantly different at *p* < 0.01.

To obtain a higher (+) borneol titer, we engineered the CbTPS1 by further structure optimization. Most terpene synthases in plants have N-terminal plastidic transit peptidases, and will be hydrolyzed after the protein is targeted to the plastid (Bohlmann et al., 1998; Zybailov et al., 2008; Rowland et al., 2015). However, this affects the catalytic activity because yeast cannot digest the transit peptide. Thus, we truncated the chloroplast transit peptide according to the prediction of ChloroP^7^; CbTPS1 was truncated at the C37 position in the N-terminus, and named t37-CbTPS1. (+)-borneol was detected by GC-MS (Figure 6A). The truncated t37-CbTPS1 showed a significant increase of (+)-borneol production to 1.53 mg⋅L^–1^ (strain MD-7) (Figure 6B).

Hamilton compared 96 *Saccharomyces cerevisiae* sequences, and analyzed the window of 100 bases around the START codon (Hamilton et al., 1987). They found that 50% of highly expressed genes use the UCU serine codon as the second triplet, which indicated that UCU following the START codon ATG could increase gene expression. Therefore, we designed two truncated proteins with ATG followed by the UCU codon. Both amino acids at positions 10 (TCC) and 32 (TCA) of CbTPS1 are serine, which is the same as the amino acid encoded by UCU, so the codon corresponding to the truncated site was mutated to TCT to increase the (+) borneol titer, resulting in t10-CbTPS1 and t32-CbTPS1, respectively. The (+) borneol titer of the two truncated proteins increased significantly. The titer of truncated t10-CbTPS1 was 1.48 mg⋅L^–1^ (strain MD-3), which was 49.33-fold higher than untruncated CbTPS1, and the titer of truncated t32-CbTPS1 was 72-fold higher than CbTPS1, up to 2.16 mg⋅L^–1^ (strain MD-5) (Figure 6B).

### Improving the (+)-Borneol Yield by Adding Kozak Sequence

The Kozak sequence is roughly the first six important nucleotides upstream of the START codon in *S. cerevisiae*, which are used for gene translation and expression. In yeast, the Kozak sequence is mostly “AAAAAA” (Hamilton et al., 1987; Li et al., 2017; Hernández et al., 2019). On the basis of truncation, yeast-specific Kozak sequence was added in front of START codon ATG of the codon-optimized CbTPS1 and three truncated proteins to increase (+)-borneol yield. The modified proteins were named CbTPS1K, t10-CbTPS1K, t32-CbTPS1K, and t37-CbTPS1K. The results showed that the yield increased at different levels after adding the Kozak sequence. The highest (+)-borneol titer was achieved in strain MD-6 containing t32-CbTPS1K, which is 96.33-fold higher than that in the strain harboring wild-type CbTPS1, producing 2.89 mg⋅L^–1^ (+)-borneol (Figure 6B).

## Discussion

Due to the insufficient supply of natural products, the role of microbial production of valuable compounds has emerged as an attractive alternative source. Microbial production is a promising choice to substitute for chemical synthesis or phytoextraction (Kirby and Keasling, 2009; Nielsen, 2019). High-efficiency gene elements are vital for metabolic engineering. In this study, we identified a (+)-bornyl diphosphate synthase (CbTPS1) from *C. burmannii* that catalyzed GPP to form (+)-borneol under the hydrolysis of CIAP. This is the first time an enzyme related to (+)-borneol synthesis was mined from *C. burmannii*, and it has the highest specificity for (+)-borneol production (Wise et al., 1998; Despinasse et al., 2017; Hurd et al., 2017; Wang et al., 2018). The *K*_*m*_ value of CbTPS1 (5.11 μM) for GPP is consistent with SBS (3.0 μM) (Wise et al., 1998) and slightly lower than other reported monoterpene synthases (13.10–26.12 μM), which indicated CbTPS1 had a higher affinity for GPP. Its *k_*cat*_/K_*m*_* (1.99 × 10^–3^ s^–1^/μM) is similar to that of other efficient and highly specific monoterpene synthases (3.55 × 10^–3^–1.23 × 10^–2^ s^–1^/μM) (Morehouse et al., 2017; Ignea et al., 2019; Dusséaux et al., 2020). Thus, it gives us an opportunity to reconstruct the (+)-borneol biosynthetic pathway in *S. cerevisiae*.

In *S. cerevisiae*, GPP is mainly produced by FPP synthase (ERG20) to serve as the intermediate product of FPP synthesis, thus, it should be consumed rapidly. As a result, when there is no engineering of ERG20, no (+)-borneol or geraniol was detected in CEN.PK2-1D. In order to increase the GPP pool, we further overexpressed all MVA pathway genes and mutated the 96F and 127N of ERG20 to obtain the strain MD, which generated the target product (+)-borneol. However, compared with the accumulation of geraniol (12.52 mg⋅L^–1^), the yield of (+)-borneol product was relatively low (0.03 mg⋅L^–1^). Thus, modified proteins were used to improve the expression and activity of CbTPS1. After steady modification, strain MD6 was obtained with the highest yield of (+)-borneol (2.89 mg⋅L^–1^). Thus, the combination of truncation and using Kozak sequence is an effective strategy for improving (+)-borneol productivity.

Though more than 20 mg⋅L^–1^ of linalool, α-terpineol, and limonene were produced in yeast (Cao et al., 2016; Zhang et al., 2019, 2020), the yields of most monoterpenes are still lower than the sesquiterpenes and diterpenes (Zebec et al., 2016; Zhao et al., 2016; Jiang et al., 2017), such as artemisinic acid (25 g⋅L^–1^) (Paddon et al., 2013) and miltiradiene (3.5 g⋅L^–1^) (Hu et al., 2020). The efficiency of forming the final product is influenced by many factors. Reduced efficiency is partially due to the high toxicity of many monoterpenes, such as pinene and limonene, to *S. cerevisiae* because they alter membrane properties or damage the cell wall (Brennan et al., 2013; Demissie et al., 2019). Two-phase extractive fermentation is usually used to alleviate the toxicity of monoterpenes (Brennan et al., 2012). We next will attempt more protein modification of CbTPS1, such as translational fusion (Ignea et al., 2019) and directed evolution of enzymes (Qu et al., 2019). In addition, optimizing the fermentation strategy, by selecting suitable solvent, and optimizing the carbon sources and fermentation parameters will further enhance production (Zhou et al., 2019). Thus, we have good reason to believe that *S. cerevisiae* could be a promising platform for a feasible, scalable, and economic route to the overproduction of (+)-borneol derivatives in the future.

## Data Availability Statement

The original contributions presented in the study are publicly available. This data can be found here: Genome Sequence Archive (Genomics, Proteomics & Bioinformatics 2017) in BIG Data Center (Nucleic Acids Res 2018), Beijing Institute of Genomics (BIG), Chinese Academy of Sciences, under accession numbers CRA003558 (http://bigd.big.ac.cn/gsa).

## Author Contributions

LH and GC conceived and designed the experiment. JG and BJ operated GC-MS. QM, HZ, and LC analyzed the data. LM provided the materials. MT purified protein. RM and PS were involved in all experimental steps and wrote this manuscript. All authors contributed to the article and approved the submitted version.

## Conflict of Interest

The authors declare that the research was conducted in the absence of any commercial or financial relationships that could be construed as a potential conflict of interest.

## Footnotes

1 https://www.novogene.com/

2 https://bigd.big.ac.cn/

3 http://www.ncbi.nlm.nih.gov/

4 http://www.ncbi.nlm.nih.gov/gorf/gorf.html

5 http://web.expasy.org/translate/

6 http://www.clcbio.com

7 http://www.cbs.dtu.dk/services/ChloroP/

8 http://www.graphpad.com

## Abbreviations

BPPS: bornyl diphosphate synthase
CIAP: calf intestinal alkaline phosphatase
DMAPP: dimethylallyl diphosphate
GC-MS: gas chromatography coupled with mass spectrometry
GPP: geranyl diphosphate
IPP: Isopentenyl diphosphate
MVA: mevalonate pathway
ORF: open reading frame
SDS-PAGE: sodium dodecyl sulfate polyacrylamide gel electrophoresis.
