# Supplementary material for: Bornyl Diphosphate Synthase From Cinnamomum burmanni and Its Application for (+)-Borneol Biosynthesis in Yeast
Source: Front Bioeng Biotechnol. 2021 Feb 11;9:631863. doi: 10.3389/fbioe.2021.631863 (PMC7905068; doi:10.3389/fbioe.2021.631863)
Supplement: Supplementary file 1 [file Data_Sheet_1.doc]

**Table S1.** Primers and sequences used in this study

| **Primer Name** | **Primer sequence (****5`→3`)** | |
| --- | --- | --- |
| **Gene cloning** | | |
| CbTPS1-F | ATGGCGTTGCAAATGACTGTTCC | |
| CbTPS1-R | TCAGGCATATCCACCATCGACAC | |
| **Vector construction (pET-32a)** | | |
| CbTPS1-F | CCATGGCTGATATCGGAATGGCGTTGCAAATGACTGTTCC | |
| CbTPS1-R | ACGGAGCTCGAATTCGGTCAGGCATATCCACCATCGACAC | |
| **Construction of (+)-borneol producing strains** | | |
| YPRCΔ15-UP-F | | ATAAAGCAGCCGCTACCAAACAGACAAGATTCAGTATG |
| YPRCΔ15-UP-R | | GCTGTGGTTTGGTAAAGCCTATAATATTATGTATACAG |
| URA3-F | | TTATAGGCTTTACCAAACCACAGCTTTTCAATTCAATTC |
| URA3-R | | AACGGACCGGGGGCATAGGGTAATAACTGATATAATTA |
| L1-F | | ATTACCCTATGCCCCCGGTCCGTTTGTTCTATACTTC |
| L1-R | | AGGCAATTTTAGAGGGGACTTCTGACTCCCCTGCTTTG |
| L6-F | | ATTGGACGAGTTCTACCTGACAGAACGGGGCTGGGAAG |
| L6-R | | AATTGTCGAAATTCCATTGTTCCGATACGCCTTGTCTCC |
| L2-F | | GACAAAGCGCCAAGGAACTGT |
| L2-R | | TAGTCCGCGAGTTGGATAGCC |
| L3-F | | AACGACGGTAGACGCCAACTAC |
| L3-R | | GCGGACTTAGTCCGTTTCTCG |
| L4-F | | CCAGACGATACAGAGGCTAAGAATAAC |
| L4-R | | CCAGACGATACAGAGGCTAAGAATAAC |
| L5-F | | CAGCCAACCAGTCAGATTAGCAGT |
| L5-R | | CGACGAACGAGATACGATAGAACG |
| YPRCΔ15-DOWN-F | | CAATGGAATTTCGACAATTATCATATTATTC |
| YPRCΔ15-DOWN-R | | GAAATGTAGATTCATTTTGTAGATTCCTATATC |
| pGAL1_GAL10-F | | CTCTGGTCTCACATCATCCGGGGTTTTTTCTCCTTGACGT |
| pGAL1_GAL10-R | | CTCTGGTCTCAATTCTTCGAATTTTCAAAAATTCTTACTT |
| ERG10-F | | CCAGGTCTCAGATGATGTCTCAGAACGTTTACATTGTATC |
| ERG10-R | | CCAGGTCTCAAGGTTCATATCTTTTCAATGACAATAGAGG |
| ERG13-F | | CCAGGTCTCAGAATATGAAACTCTCAACTAAACTTTGTTG |
| ERG13-R | | CCAGGTCTCACGATTTATTTTTTAACATCGTAAGATCTTC |
| ERG10-ERG13 cassette-F | | CCCCGGTCCGTTTGTTCTATACTTC |
| ERG10-ERG13 cassette-R | | TAGTCCGCGAGTTGGATAGCCCGAG |
| tHMG1-F1 | | CCAGGTCTCAGATGATGGCTGCAGACCAATTGGTGAAAAC |
| tHMG1-R1 | | CCAGGTCTCAAGGTTTAGGATTTAATGCAGGTGACGGACC |
| tHMG1-F2 | | CCAGGTCTCAGAATATGGCTGCAGACCAATTGGTGAAAAC |
| tHMG1-R2 | | CCAGGTCTCACGATTTAGGATTTAATGCAGGTGACGGACC |
| tHMG1-tHMG1 cassette-F | | GACAAAGCGCCAAGGAACTGTAATAT |
| tHMG1-tHMG1 cassette-R | | GCGGACTTAGTCCGTTTCTCGGCTAT |
| ERG12-F | | CCAGGTCTCAGAATATGTCATTACCGTTCTTAACTTCTGC |
| ERG12-R | | CCAGGTCTCACGATTTATGAAGTCCATGGTAAATTCGTGT |
| tHMG1-ERG12 cassette-F | | AACGACGGTAGACGCCAACTACGCTG |
| tHMG1-ERG12 cassette-R | | AGGTTCCAACTGCTCTTACTGTAGTC |
| ERG8-F | | CCAGGTCTCAGATGATGTCAGAGTTGAGAGCCTTCAGTGC |
| ERG8-R | | CCAGGTCTCAAGGTTTATTTATCAAGATAAGTTTCCGGAT |
| ERG19-F | | CCAGGTCTCAGAATATGACCGTTTACACAGCATCCGTTAC |
| ERG19-R | | CCAGGTCTCACGATTTATTCCTTTGGTAGACCAGTCTTTG |
| ERG8-ERG19 cassette-F | | CCAGACGATACAGAGGCTAAGAATAAC |
| ERG8-ERG19 cassette-R | | CGACGAACGAGATACGATAGAACGTT |
| IDI1-F | | CCAGGTCTCAGATGATGACTGCCGACAACAATAGTATGC |
| IDI1-R | | CCAGGTCTCAAGGTTTATAGCATTCTATGAATTTGCCTG |
| ERG20^F96W-N127W^-F | | CCAGGTCTCAGAATATGGCTTCAGAAAAAGAAATTAGGA |
| ERG20^F96W-N127W^-R | | CCAGGTCTCACGATCTATTTGCTTCTCTTGTAAACTTTG |
| IDI1-ERG20^F96W-N127W^ cassette-F | | CAGCCAACCAGTCAGATTAGCAGTAT |
| IDI1-ERG20^F96W-N127W^ cassette-R | | TTCCGATACGCCTTGTCTCCAACTCAAT |
| **Vector construction (pESC-LEU)** | | |
| CbTPS1-F’ | AGGAGAAAAAACCCCGATGGCCTTGCAAATGACCGTCC | |
| CbTPS1-R’ | GTGAGTCGTATTACGGTCAAGCGTAACCACCGTCAACGC | |
| CbTPS1-F’K | AGGAGAAAAAACCCCGAAAAAAATGGCCTTGCAAATGACCGTCC | |
| CbTPS1-F10 | AGGAGAAAAAACCCCGATGTCTTCCTTCTTGCCAAACCCAAG | |
| CbTPS1-F10K | AGGAGAAAAAACCCCGAAAAAAATGTCTTCCTTCTTGCCAAACCCAAG | |
| CbTPS1-F32 | AGGAGAAAAAACCCCGATGTCTAAGCACATCTCTTGCTCTACCAC | |
| CbTPS1-F32K | AGGAGAAAAAACCCCGAAAAAAATGTCTAAGCACATCTCTTGCTCTACCAC | |
| CbTPS1-F37 | AGGAGAAAAAACCCCGATGTGCTCTACCACCACTCCAACCTACTC | |
| CbTPS1-F37K | AGGAGAAAAAACCCCGAAAAAAATGTGCTCTACCACCACTCCAACCTACTC | |
| **Sequence Name** | **Sequence (5`→3`)** | |
| L1-TPI1-PGI-L2 | CCCCGGTCCGTTTGTTCTATACTTCTCTCTGCTATACCTACAAGCAAGGTAATCGGAAGTAGTATTACGCAGGAATATCCCGCGCGAAGCTACAATTTTTGGACTCCAACGTCAAAGCAGGGGAGTCAGAAGTCCCCTCTAAAATTGCCTCTATATAACAGTTGAAATTTGGATAAGAACATCTTCTCAACGCGAAAATGACGCCTCCAGTGAAAAAACATAAACTTTCAATGCAGTCTTCGGTACACTTATGAGTAACCCATATAGAGATCGTACACATTTTACAAGGATTTAGAGACAAAGTAATATTCTTCGTTGATATAGAGGTGTTCAATTGTTAAATGCTTTTCTTCTTTTTATTAGAAAAAGCGCCTTGCTTTTTGTTGTATCACTTGTAAATCTACCGTCCCTTACAAGAACATTCACGAAATTTAAGTGGCTCAGAATGAAAAAGAAACAATATAAAAAAGCTTTCCGTAGTCATCAATTTATTTTACATAACACTAGATATAAAGAAAAGAAGATAATATTTTTATATAATTATATTAATCAGGTTGAGACCGGTCTCAATCGAACAAATCGCTCTTAAATATATACCTAAAGAACATTAAAGCTATATTATAAGCAAAGATACGTAAATTTTGCTTATATTATTATACACATATCATATTTCTATATTTTTAAGATTTGGTTATATAATGTACGTAATGCAAAGGAAATAAATTTTATACATTATTGAACAGCGTCCAAGTAACTACATTATGTGCACTAATAGTTTAGCGTCGTGAAGACTTTATTGTGTCGCGAAAAGTAAAAATTTTAAAAATTAGAGCACCTTGAACTTGCGAAAAAGGTTCTCATCAACTGTTTAAAAGGAGGATATCAGGTCCTATTTCTGACAAACAATATACAAATTTAGTTTCAAAGATGAATCAGTGCGCGAAGGACATAACTCATGAAGCCTCCAGTATACCGACAAAGCGCCAAGGAACTGTAATATATAGCTACGCCCTATCTGGACGATTGGGCGACTTTTACGTACGGTTGCTCAATTCCTACGCAACTTAATATATTTTGCAACGGTTAAATCGGCTTGAAGCTCGGGCTATCCAACTCGCGGACTA | |
| L2-ADH1-CYC1-L3 | GACAAAGCGCCAAGGAACTGTAATATATAGCTACGCCCTATCTGGACGATTGGGCGACTTTTACGTACGGTTGCTCAATTCCTACGCAACTTAATATATTTTGCAACGGTTAAATCGGCTTGAAGCTCGGGCTATCCAACTCGCGGACTATCGGCATGCCGGTAGAGGTGTGGTCAATAAGAGCGACCTCATGCTATACCTGAGAAAGCAACCTGACCTACAGGAAAGAGTTACTCAAGAATAAGAATTTTCGTTTTAAAACCTAAGAGTCACTTTAAAATTTGTATACACTTATTTTTTTTATAACTAGGTTGAGACCGGTCTCAATCGTCATGTAATTAGTTATGTCACGCTTACATTCACGCCCTCCCCCCACATCCGCTCTAACCGAAAAGGAAGGAGTTAGACAACCTGAAGTCTAGGTCCCTATTTATTTTTTTATAGTTATGTTAGTATTAAGAACGTTATTTATATTTCAAATTTTTCTTTTTTTTCTGTACAGACGCGTGTACGCATGTAACATTATACTGAAAACCTTGCTTGAGAAGGTTTTGGGACGCTCGAAGGCTTTAATTTGCAACGACGGTAGACGCCAACTACGCTGACAGACCGATTTGTTTAAGATTAGAAGATTTTTAGCCGCGCCGCAATCGGAACCAGCAAACTCAATTCTGGGAACAGTTTAAAATACTAGTAATTACGATAGCCGAGAAACGGACTAAGTCCGC | |
| L3-FBA1-PDC1-L4 | AACGACGGTAGACGCCAACTACGCTGACAGACCGATTTGTTTAAGATTAGAAGATTTTTAGCCGCGCCGCAATCGGAACCAGCAAACTCAATTCTGGGAACAGTTTAAAATACTAGTAATTACGATAGCCGAGAAACGGACTAAGTCCGCGCTATCAAAAACGATAGATCGATTAGGATGACTTTGAAATGACTCCGCAGTGGACTGGCCGTTAATTTCAAGCGTGAGTAAAATAGTGCATGACAAAAGATGAGCTAGGCTTTTGTAAAAATATCTTACGTTGTAAAATTTTAGAAATCATTATTTCCTTCATATCATTTTGTCATTGACCTTCAGAAGAAAAGAGCCGACCAATAATATAAATAAATAAATAAAAATAATATTCCATTATTTCTAAACAGATTCAATACTCATTAAAAAACTATATCAATTAATTTGAATTAACAGGTTGAGACCGGTCTCAATCGGCGATTTAATCTCTAATTATTAGTTAAAGTTTTATAAGCATTTTTATGTAACGAAAAATAAATTGGTTCATATTATTACTGCACTGTCACTTACCATGGAAAGACCAGACAAGAAGTTGCCGACAGTCTGTTGAATTGGCCTGGTTAGGCTTAAGTCTGGGTCCGCTTCTTTACAAATTTGGAGAATTTCTCTTAAACGATATGTATATTCTTTTCGTTGGAAAAGATGTCTTCCAAAAAAAAAACCGATGAATTAGTGGAACCAAGGAAAAAAAAAGAGGTATCCTTGATTAAGGAACACCAGACGATACAGAGGCTAAGAATAACGCAGATAATCGCTCTAACGAAACGTACTAAAAGATTTCTTTTGAAGTAACTAGATACCCTGGTCTTATACTAGGTATCTTTGTCAGAAACGGCCTAAGACTACAGTAAGAGCAGTTGGAACCT | |
| L4-RPS2-TDH1-L5 | CCAGACGATACAGAGGCTAAGAATAACGCAGATAATCGCTCTAACGAAACGTACTAAAAGATTTCTTTTGAAGTAACTAGATACCCTGGTCTTATACTAGGTATCTTTGTCAGAAACGGCCTAAGACTACAGTAAGAGCAGTTGGAACCTTAAAATTTTGATCTATTGTAGTCGCCTAATCTTGCTTCTCTTGCTGAATAAATAGCGCTATCCGCTGTACCGTACCAAGAGTGACCTACAGCGTTTGATAGGCAAAAGAGTTAAGGACAGACTGGGTTTCAATCAAAATAAATGCCAAAAGGATTTGACTTTATATTTGATAATGCTCACTCTAAACGGAAGGCACATGCATCGCATAATTGGAACGAGAAATAAAGTGTTATTATAAGTGTACAAAATTATTTAATGGTTGATTATAAAAAACTATTTTATAATTTGTAGACAACAAGCAGGTTGAGACCGGTCTCAATCGATAAAGCAATCTTGATGAGGATAATGATTTTTTTTTGAATATACATAAATACTACCGTTTTTCTGCTAGATTTTGTGAAGACGTAAATAAGTACATATTACTTTTTAAGCCAAGACAAGATTAAGCATTAACTTTACCCTTTTCTCTTCTAAGTTTCAATACTAGTTATCACTGTTTAAAAGTTATGGCGAGAACGTCGGCGGTTAAAATATATTACCCTGAACGTGGTGAATTGAAGTTCTAGGATGGTTTAAAGATTTTTCCTTTTTGGGAAATAAGTAAACAATATATTGCTGCCTTCAGCCAACCAGTCAGATTAGCAGTATATAGGCTGCGAGTTACTAGCGGGCAAAATCTGGAGTTATCGCCTAAAGCGTTATCTGATCCCTATTCGGCAGCAGTTTGGTCGGTTTGAGTAGTCAATAACGTTCTATCGTATCTCGTTCGTCG | |
| L5-CCW12-RPL9A-L6 | CAGCCAACCAGTCAGATTAGCAGTATATAGGCTGCGAGTTACTAGCGGGCAAAATCTGGAGTTATCGCCTAAAGCGTTATCTGATCCCTATTCGGCAGCAGTTTGGTCGGTTTGAGTAGTCAATAACGTTCTATCGTATCTCGTTCGTCGTGTTCTAGTGTGTTTATATTATCCTTGGCTAAGAGGCACTGCGTATACTTCAAGGTACGCCTGTGTTTTGAAAAAAAACAACAGTAAAATAGGAACTCCGCGAGGTTCAGGAACCTGAAACAAAATCAATAAAAACATTATATGCGTTTCGAACAAAATTAAAGAAAAAGAATAAATATAGATTAAAAAAAAAAAGAAGAAATTAAAAGAATTTCTACTAAATCCCAATTGTTATATATTTGTTAAATGCCAAAAAAGTTTATAAAAAATTTAGAATGTATAAATAATAATAAACTAAGTAGGTTGAGACCGGTCTCAATCGGCTTTTTATTTGATTTTGTGACATTTTCTTTAAAGTAGCAGAAAAAATAATTACATATAAAAAACTTTGTATATCTAACATATAATATAATTGAACCGATACGAAAATAAAAGTACCGATAATATGTCAACTTTTTGTTTTTTGTTGCCGTTTTATTACGTGCACTAATACATATACATGCGTATATAAATATACATAACTCTTGAGGGGTTTTGTTTCTATCTATTGGACGAGTTCTACCTGACAGAACGGGGCTGGGAAGAGAAAGATCCTAGCTGGCGCCTATACTACTTCCAAGAACCATTCCCCAGCTTATCAAGAGTTTTCTCGTTCAGCTACCGACTGTATTGAGTTGGAGACAAGGCGTATCGGAA | |

* The underlined bases represent not only the corresponding endonuclease sequences, but also the overlapping sequences with the corresponding vector, which are designed following the protocol of the *pEASY*®-Uni Seamless Cloning and Assembly Kit (TransGen Biotech). F, forward; R, reverse.


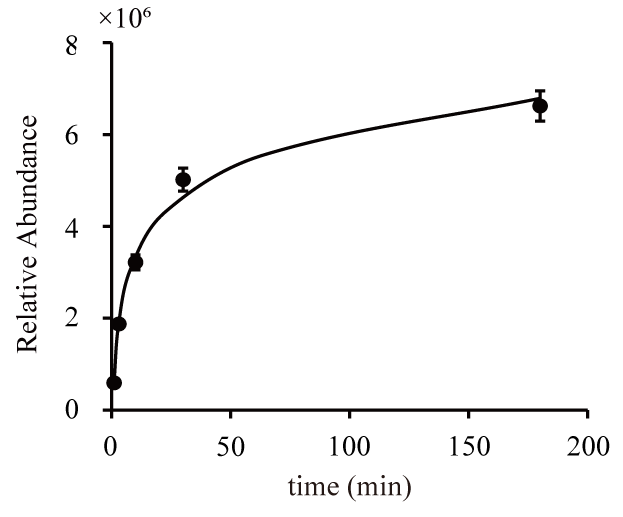


**Fig. S1**. Time course assay of CbTPS1 activity.


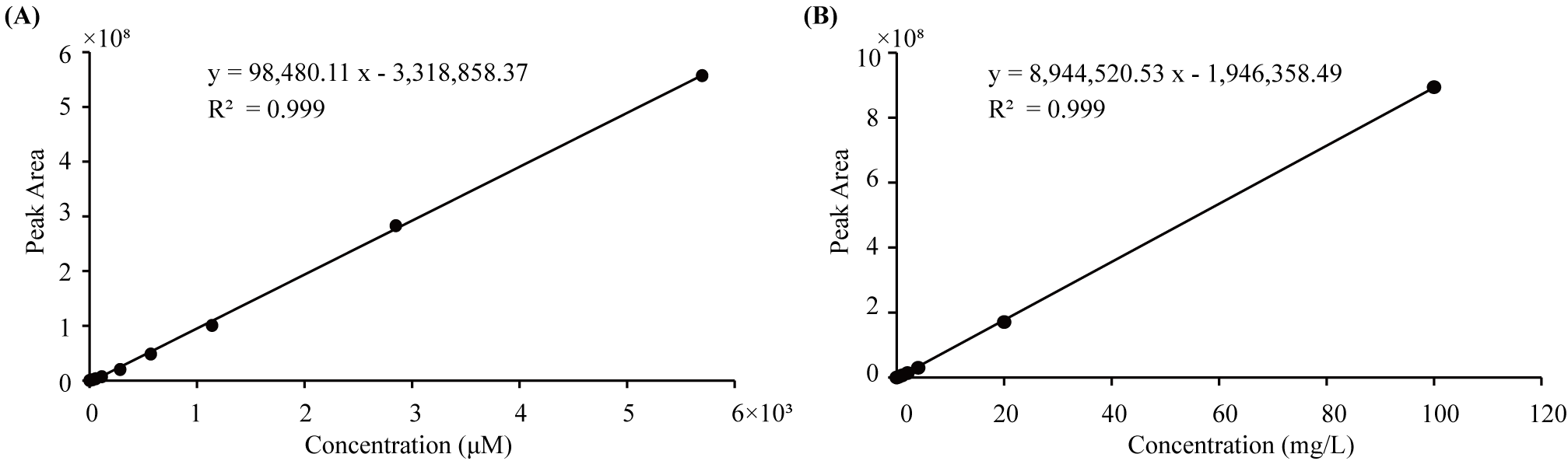


**Fig. S2**. Calibration curve used in estimation of geraniol and (+)-borneol.(A) authentic standard geraniol, (B) authentic standard (+)-borneol.
